# Elevated CO_2_ concentration induces photosynthetic down-regulation with changes in leaf structure, non-structural carbohydrates and nitrogen content of soybean

**DOI:** 10.1186/s12870-019-1788-9

**Published:** 2019-06-13

**Authors:** Yunpu Zheng, Fei Li, Lihua Hao, Jingjin Yu, Lili Guo, Haoran Zhou, Chao Ma, Xixi Zhang, Ming Xu

**Affiliations:** 1 School of Water Conservancy and Hydropower, Hebei University of Engineering, Handan, 056038 China; 2 School of Agro-Grassland Science, Nanjing Agricultural University, Nanjing, 210095 People’s Republic of China; 3 Department of Biology, University of Pennsylvania, Philadelphia, PA 19104 USA; 4 Key Laboratory of Geospatial Technology for the Middle and Lower Yellow River Regions, College of Environment and Planning, Henan University, Kaifeng, 475004 China; 5 Center for Remote Sensing and Spatial Analysis, Department of Ecology, Evolution and Natural Resources, Rutgers University, 14 College Farm Road, New Brunswick, NJ 08901 USA

**Keywords:** CO_2_ enhancement, Down regulation, Non-structural carbohydrates, N availability, Stomatal traits, Soybean crops

## Abstract

**Background:**

Understanding the mechanisms of crops in response to elevated CO_2_ concentrations is pivotal to estimating the impacts of climate change on the global agricultural production. Based on earlier results of the “doubling-CO_2_ concentration” experiments, many current climate models may overestimate the CO_2_ fertilization effect on crops, and meanwhile, underestimate the potential impacts of future climate change on global agriculture ecosystem when the atmospheric CO_2_ concentration goes beyond the optimal levels for crop growth.

**Results:**

This study examined the photosynthetic response of soybean (*Glycine max* (L.) Merr.) to elevated CO_2_ concentration associated with changes in leaf structure, non-structural carbohydrates and nitrogen content with environmental growth chambers where the CO_2_ concentration was controlled at 400, 600, 800, 1000, 1200, 1400, 1600 ppm. We found CO_2_-induced down-regulation of leaf photosynthesis as evidenced by the consistently declined leaf net photosynthetic rate (*A*_n_) with elevated CO_2_ concentrations. This down-regulation of leaf photosynthesis was evident in biochemical and photochemical processes since the maximum carboxylation rate (*V*_cmax_) and the maximum electron transport rate (*J*_max_) were dramatically decreased at higher CO_2_ concentrations exceeding their optimal values of about 600 ppm and 400 ppm, respectively. Moreover, the down-regulation of leaf photosynthesis at high CO_2_ concentration was partially attributed to the reduced stomatal conductance (*G*_s_) as demonstrated by the declines in stomatal density and stomatal area as well as the changes in the spatial distribution pattern of stomata. In addition, the smaller total mesophyll size (palisade and spongy tissues) and the lower nitrogen availability may also contribute to the down-regulation of leaf photosynthesis when soybean subjected to high CO_2_ concentration environment.

**Conclusions:**

Down-regulation of leaf photosynthesis associated with the changes in stomatal traits, mesophyll tissue size, non-structural carbohydrates, and nitrogen availability of soybean in response to future high atmospheric CO_2_ concentration and climate change.

## Background

It is well known that human activities have dramatically increased atmospheric concentrations of greenhouse gases [1, 2], particularly the elevated atmospheric carbon dioxide concentration due to fossil fuel combustion and land use change following the nineteenth century industrial revolution [3–5]. The most recently released report by the Inter-governmental Panel on Climate Change (IPCC) [6] showed that global atmospheric CO_2_ concentration has been dramatically increased from 280 ppm (the pre-industrial level) to over 400 ppm (the present level) with the growth rate of CO_2_ concentration by ∼1.0 ppm per year [6], and may even be over 1000 ppm at the end of this century [7]. The elevated atmospheric CO_2_ concentration may lead to drastic impacts on the structure and function of natural and managed ecosystems [8–12].

Plant responses to elevated CO_2_ concentration are fundamentally mediated by leaf photosynthesis, which is closely associated with the changes in leaf structure, chemical composition and carbon balance depending on plant species and/or functional types [13–15]. Many previous studies have shown that elevated CO_2_ generally stimulated the net photosynthetic rate (*A*_n_) of plants, namely “CO_2_ fertilization effect”, especially for the C_3_ species, because the ribulose-1, 5-bisphophate carboxylase/oxygenase (Rubisco) of C_3_ plants is not CO_2_-saturated at the current atmospheric CO_2_ concentration [14, 16–21]. Meanwhile, the enhanced *A*_n_ may also be resulted from the reduced photorespiration and dark respiration and enhanced carboxylation efficiency under high CO_2_ concentrations [22–25]. However, other studies reported that the *A*_n_ was not marginally enhanced and even declined when plants exposed to long-term elevated CO_2_ concentrations [26–28]. For example, Kanemoto [29] found that leaf photosynthesis of soybean plants was substantially decreased with elevating CO_2_ concentration from about 400 ppm to 1000 ppm for 27 days of treatment. This down-regulation of *A*_n_ may be attributed to the lower Rubisco concentration and activity [30–34] or/and the source-sink imbalance due to leaf carbohydrates accumulation under elevated CO_2_ concentration [29, 35–38]. In addition, the down-regulation of *A*_n_ at high CO_2_ concentration may also be caused by the decline of stomatal conductance [4, 39–46]. Xu [47] found that the decline in biomass of winter wheat at high CO_2_ concentration might be attributed to the decrease of *G*_s_ mainly due to the reduction in stomatal length and stomatal density.

In addition to physiological traits, leaf structural and biochemical characteristics may also play a pivotal role in plant response to high CO_2_ concentration [48–50]. Elevated CO_2_ concentration usually generates greater leaf thickness and total mesophyll size, which closely correlated to leaf photosynthetic rate [51–54]. Previous studies have shown that elevated CO_2_ concentration increased the leaf thickness and mesophyll cross-section area, which was mainly attributable to greater cell expansion rather than enhanced cell division due to the increase of carbohydrate substrate availability [55–57]. The thicker mesophyll tissue and larger cell volume may provide more space for accommodating chloroplasts and more intercellular surface area for leaf gas exchange [42, 58–60]. Meanwhile, elevated CO_2_ concentration may also change leaf biochemical compositions including the non-structural carbohydrates and nitrogen concentration (N), which play an important role in controlling over the responses of plants and/or ecosystems to rising atmospheric CO_2_ levels [17, 61, 62]. Understanding the mechanisms of leaf structure and biochemistry in response to high CO_2_ concentration is critical for assessing the changes in leaf functional traits and thus ecosystem functioning under future global change.

Several previous studies have documented that different plants features with different optimal CO_2_ concentrations for plant growth [47, 63] and thus plants with high optimal CO_2_ concentrations will suffer less from climate change and meanwhile benefit the most from the CO_2_ fertilization effect due to high nitrogen and water use efficiency [24]. Exploring the mechanisms of CO_2_ fertilization effect on crops is critical to estimating global agriculture yield under climatic change [64]. Numerous studies have investigated CO_2_ fertilization effect primarily focusing on the impact of twofold current [CO_2_] on plants with doubling CO_2_ concentration experiment [64–66], which normally increased CO_2_ concentration from 300 to 400 ppm to the projected atmospheric CO_2_ concentration of 600–800 ppm at the end of the next century [6, 67]. However, the atmosphere CO_2_ concentration has covered a much wider range throughout geological time scales with an estimated value of 6000 ppm during the Paleozoic Era about 500 million years ago [24]. To our knowledge, few studies have examined the responsible mechanism of *A*_n_ associated with changes in leaf structure, non-structural carbohydrates and nitrogen content of soybean (*Glycine max* (L.) Merr.), the fourth important crop species in the world under higher CO_2_ concentrations beyond the twofold current CO_2_ concentration of 800 ppm. Therefore, we conducted this experiment with environmental growth chambers controlling multiple high CO_2_ levels from 400 ppm to 1600 ppm to test the following hypotheses:

1. Leaf photosynthesis is down-regulated at higher CO_2_ concentrations beyond the optimal atmospheric CO_2_ concentration for the growth of soybean (HY1).
2. This down-regulation of leaf photosynthesis may attribute to the declines in biochemical and photochemical efficiency such as the maximum carboxylation rate (*V*_cmax_) and the maximum electron transport rate (*J*_max_) (HY2).
3. The CO_2_-induced stomatal closure and irregular distribution pattern of stomata on soybean leaves will partially explain the down-regulation of leaf photosynthesis under high CO_2_ concentrations (HY3).
4. Changes in leaf mesophyll anatomy and chemical composition may also play essential roles in the down-regulation processes of leaf photosynthesis when soybean subjected to elevated atmospheric CO_2_ concentrations (HY4).

## Results

### CO_2_ effects on leaf photosynthesis, stomatal conductance, water use efficiency, and dark respiration

**Fig. 1:**
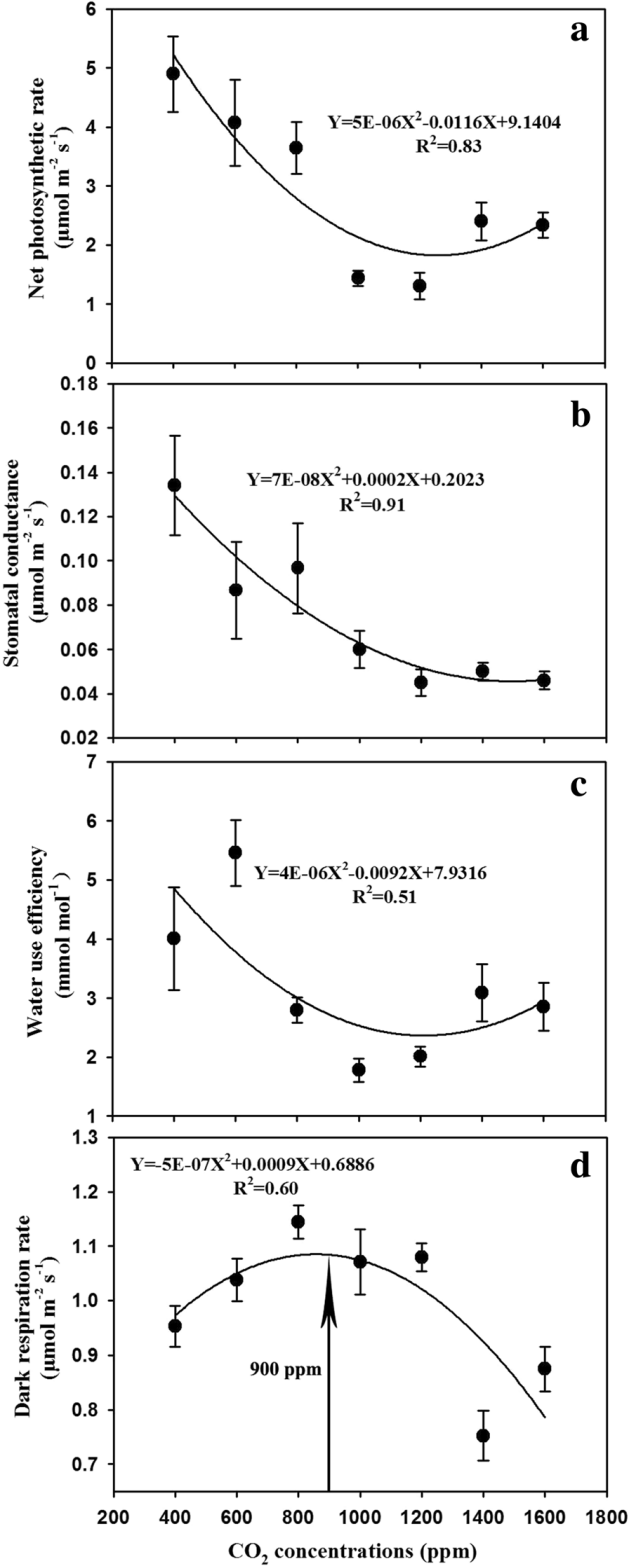
Effects of elevated CO_2_ concentrations on (**a**) leaf net photosynthesis rate (*A*_n_), (**b**) stomatal conductance (*G*_s_), (**c**) intercellular CO_2_ concentration (*C*_i_), and (**d**) dark respiration rate (*R*_d_). Parameters are measured at ambient or elevated CO_2_ of their growing condition for each treatment and values given are mean ± standard deviation for n = 5 leaves. The arrow indicates the optimal CO_2_ concentration for leaf *R*_d_ of soybean plants

We found a negative quadratic relationship between leaf photosynthesis and CO_2_ concentration (R^2^ = 0.83) with the minimum leaf photosynthesis occurred at the CO_2_ concentration of 1200 ppm (Fig. 1a). Similar with the leaf photosynthesis, elevated CO_2_ concentrations resulted in non-linear decrease in stomatal conductance, which followed a quadratic relationship (R^2^ = 0.91) with the minimum value occurring around 1200 ppm (Fig. 1b). Meanwhile, a quadratic equation can also be used to describe the relationship (R^2^ = 0.51) between the leaf-level water use efficiency (*WUE*) and the CO_2_ concentration (Fig. 1c). However, the leaf dark respiration rate demonstrated a bell-shaped curve (R^2^ = 0.60) peaking at 900 ppm in relation to CO_2_ concentration (Fig. 1d).

### CO_2_ effects on *V*_cmax_, *J*_max_, and the *V*_cmax_ /*J*_max_ ratio

**Fig. 2:**
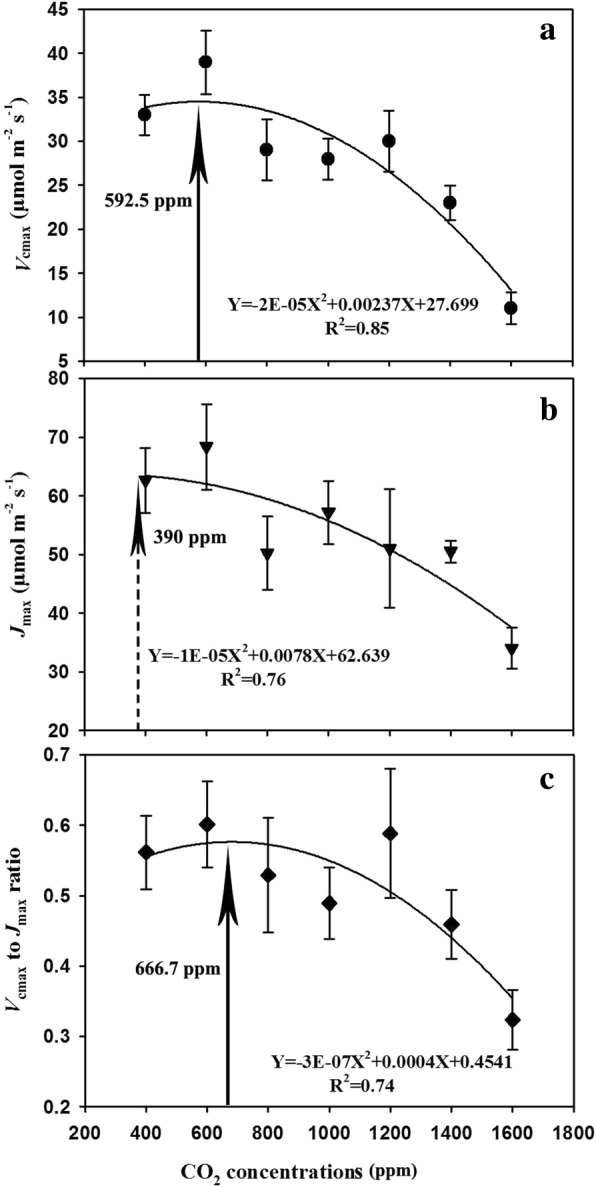
Effects of elevated CO_2_ concentrations on the *V*_cmax_ (a), *J*_max_ (b) and *V*_cmax_/*J*_max_ ratio of soybean plants

Both the maximum carboxylation rate (*V*_cmax_) and the *V*_cmax_/*J*_max_ ratio in response to increasing CO_2_ concentration featured bell-shaped curves, peaking at 592.5 ppm (Fig. 2a) and 666.7 ppm (Fig. 2c), respectively. However, the increase in CO_2_ concentration led to a non-linear decline in the maximum electron transport rate (*J*_max_) with the maximum value occurring around 390 ppm (Fig. 2b). These relationships of *V*_cmax_, *J*_max_, and *V*_cmax_/*J*_max_ ratio in relation to CO_2_ concentration could be described by quadratic equations with R^2^ values of 0.85. 0.76, and 0.74, respectively (Fig. 2).

### CO_2_ effects on morphological traits and spatial distribution pattern of stomata

**Fig. 3:**
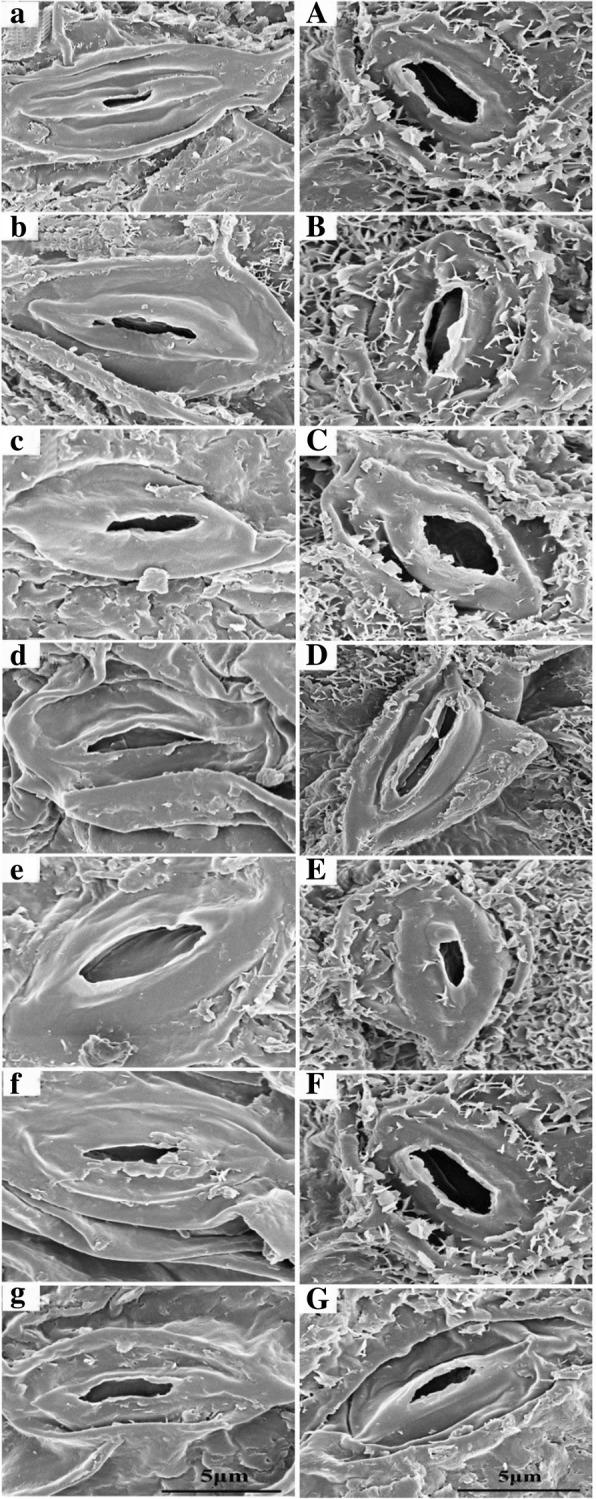
Changes in the morphological traits of stomata on the adaxial leaf surface (**a**-**g**) and abaxial leaf surface (**A**-**G**) of soybean leaves grown at CO_2_ concentrations of 400, 600, 800, 1000, 1200, 1400 and 1600 ppm observed with scanning electron microscopy. Bars = 5 μm

We found that the stomatal area was substantially enhanced by 37% on the adaxial surfaces enhancing CO_2_ concentration from 400 to 1200 ppm (*p* = 0.03), although stomatal length, width, perimeter and shape index were barely affected by elevated CO_2_ concentration (*p* > 0.05; Table 1; Fig. 3). Our results also showed that elevated CO_2_ concentration significantly decreased stomatal area index on both the adaxial and abaxial leaf surfaces except for increasing CO_2_ concentration from 400 to 600 ppm, where the stomatal area index on the abaxial side was marginally increased by 15% and reached its maximum value at 600 ppm (Table 1; Fig. 3). By contrast, the stomatal area index on the adaxial surface was significantly decreased by about 60% with the increase of CO_2_ concentration from 400 to 600 ppm and reached its minimum value at 600 ppm (Table 1; Fig. 3). Moreover, our results also showed that the stomatal density on the adaxial side was decreased by about 57% (*p* = 0.01), 61% (*p* = 0.013), 38% (*p* = 0.025), 32% (*p* = 0.026) and 48% (*p* = 0.003) with increasing CO_2_ concentration to 600, 800, 1200, 1400, and 1600 ppm, respectively (Table 1; Fig. 3). However, elevating CO_2_ concentration from 400 to 600 ppm made the stomatal density on the abaxial sides increased 21% (Table 1; Fig. 3). In addition, we also found the interactive effect of leaf surface and CO_2_ concentration on the stomatal density (*p* = 0.009) and stomatal area (*p* = 0.006; Table 2).

**Table 1:** Effects of CO_2_ concentrations on the stomatal density and morphological traits of individual stoma

Stomatal parameters	CO_2_ concentration (ppm)							
	400	600	800	1000	1200	1400	1600	*p* value
Adaxial surface								
Stomatal length (μm)	9.4 ± 1.3 (a)	9. 7 ± 1.6 (a)	9.6 ± 0.6 (a)	9.6 ± 0.8 (a)	9.0 ± 2.0 (a)	9.50 ± 0.5 (a)	9.8 ± 1.6 (a)	*p* > 0.05
Stomatal width (μm)	2.2 ± 0.9 (a)	1.8 ± 0.4 (a)	2.6 ± 0.5 (a)	1.7 ± 0.2 (a)	2.7 ± 0.5 (a)	2.4 ± 0.5 (a)	2.5 ± 0.7 (a)	*p* > 0.05
Stomatal area (μm^2^)	92.6 ± 16.2 (b)	88.7 ± 9.6 (b)	105.4 ± 5.2 (ab)	83.3 ± 9.0 (b)	126.4 ± 24.1 (a)	100.3 ± 17.2 (b)	103.9 ± 11.0 (ab)	*p* = 0.03
Stomatal perimeter (μm)	40.7 ± 0.5 (ab)	45.0 ± 1.0 (ab)	41.0 ± 0.1 (ab)	37.9 ± 0.2 (b)	46.2 ± 0.4 (a)	40.5 ± 0.3 (ab)	41.2 ± 0.27 (ab)	*p >* 0.05
Stomatal density (No./mm^2^)	16.8 ± 8.9 (a)	7.3 ± 4.9 (c)	6.6 ± 4.7 (bc)	17.8 ± 5.3 (a)	10.4 ± 6.4 (bc)	11.4 ± 6.3 (b)	8.7 ± 3.5 (b)	*p* ***=*** 0.04
Stomatal shape index (%)	24.0 ± 1.3 (a)	20.4 ± 4.7 (b)	25.0 ± 0.2 (a)	24.1 ± 0.2 (a)	24.3 ± 2.4 (a)	24.6 ± 0.6 (a)	24.7 ± 0.7 (a)	*p* > 0.05
Stomatal area index (%)	9.4 ± 1.7 (a)	3.9 ± 0.4 (d)	4.2 ± 0.3 (d)	9.0 ± 1.0 (a)	7.9 ± 1.5 (ab	6.9 ± 1.2 (bc)	5.4 ± 0.6 (cd)	*p* < 0.001
Abaxial surface								
Stomatal length (μm)	9.6 ± 1.7 (ab)	9.8 ± 0.6 (ab)	9.5 ± 0.8 (ab)	10.2 ± 0.7 (a)	8.9 ± 0.6 (b)	9.4 ± 0.2 (ab)	9.2 ± 0.1 (ab)	*p* > 0.05
Stomatal width (μm)	3.1 ± 0.9 (a)	3.1 ± 0.3 (a)	3.2 ± 0.3 (a)	2.9 ± 0.4 (a)	3.0 ± 0.4 (a)	3.1 ± 0.2 (a)	2.8 ± 0.1 (a)	*p* > 0.05
Stomatal area (μm^2^)	133.1 ± 0.5 (a)	123.9 ± 16.5 (ab)	108.9 ± 13.8 (bc)	102.3 ± 6.1 (c)	109.3 ± 7.5 (bc)	113.9 ± 9.2 (abc)	110.1 ± 17.8 (bc)	*p* > 0.05
Stomatal perimeter (μm)	42.8 ± 5.5 (a)	44.6 ± 2.8 (a)	41.1 ± 3.1 (a)	40.7 ± 1.4 (a)	40.7 ± 2.0 (a)	42.6 ± 1.7 (a)	41.8 ± 3.3 (a)	*p* > 0.05
Stomatal density (No./mm^2^)	101.3 ± 20.1 (abc)	128.3 ± 18 (a)	95.7 ± 10.8 (bc)	92 ± 17.0 (c)	104.8 ± 9.7 (abc)	84.2 ± 9.2 (c)	111.4 ± 17.8 (ab)	*p* > 0.05
Stomatal shape index (%)	25.3 ± 0.1 (a)	24.9 ± 0.1 (a)	25.2 ± 0.4 (a)	24.9 ± 0.1 (a)	25.1 ± 0.5 (a)	25.0 ± 0.4 (a)	25.1 ± 0. (a)	*p* > 0.05
Stomatal area index (%)	81.9 ± 0.3 (b)	96.3 ± 12.8 (a)	63.2 ± 8.0 (cd)	57.1 ± 3.4 (d)	69.5 ± 4.8 (bcd))	58.2 ± 4.72 (d)	74.8 ± 12.1 (bc)	*p* < 0.001
Adaxial/abaxial ratio	0.23 ± 0.023 (ab)	0.12 ± 0.022 (c)	0.13 ± 0.042 (c)	0.26 ± 0.002(a)	0.21 ± 0.018 (b)	0.20 ± 0.013 (b)	0.09 ± 0.024 (c)	*p* < 0.001

**Table 2:** Effects of CO_2_ concentrations on the stomatal parameters at different leaf surfaces of soybean

Parameters	SD (No. mm^−2^)	SL (mm)	SW (mm)	SA (mm^2^)	SP (mm)	SAI (%)	SSI (%)
CO_2_	0.149	0.531	0.352	0.102	0.910	0.194	0.130
Leaf surface	< 0.001	< 0.001	< 0.001	< 0.001	0.805	0.933	0.002
CO_2_ × Leaf surface	0.009	0.158	0.350	0.006	0.269	0.323	0.290

Note: *SD* is stomatal density, *SL* is stomatal length, *SW* is stomatal width, *SA* is stomatal area, *SP* is stomatal perimeter, *SAI* is stomatal area index, and *SSI* is stomatal shape index

**Fig. 4:**
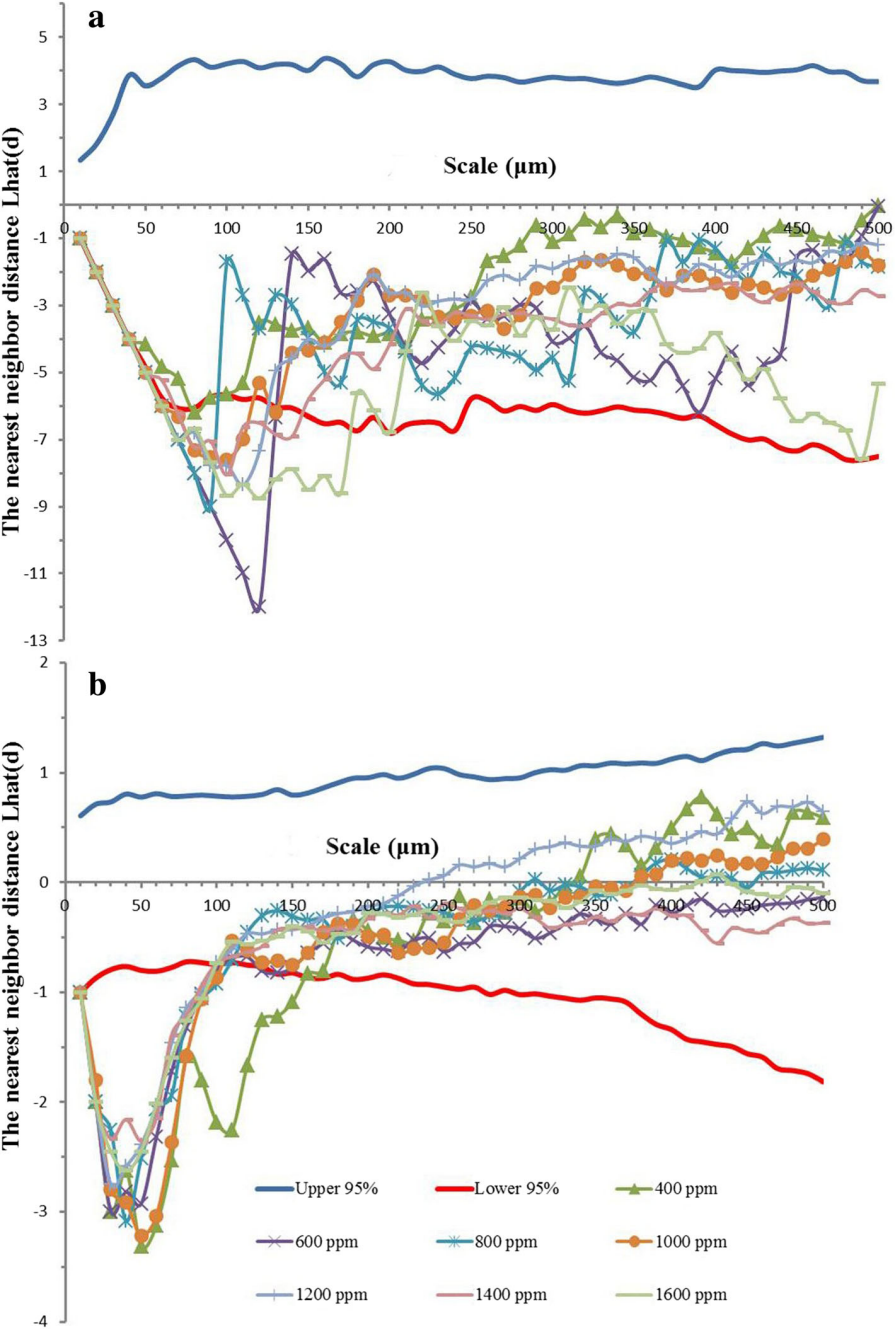
The spatial distribution pattern of stomata on the adaxial surface (**a**) and abaxial surface (**b**) of soybean leaves under elevated CO_2_ concentrations. The upper and lower 95% boundaries were obtained by Monte Carlo simulation of 100 replicates

Elevated CO_2_ concentration not only changed the morphological traits of individual stoma but also affected stomatal distribution on soybean leaves. We found that the spatial distribution pattern of stomata was highly scale-dependent with regular patterns at small scales of about 70–170 μm (below the lower 95% envelope) and random patterns at larger scales up to 200 μm (between the upper and lower 95% envelope) on both leaf surfaces (Fig. 4). Increasing CO_2_ concentration from 400 to 600 ppm caused the stomatal distribution to become more regular at small scales on the adaxial surface as evidenced by the decrease of Lhat (d) value from − 1.69 to − 12.00. However, the stomata on the abaxial surfaces tend to be less regular than those on the adaxial surface because the abaxial surface had higher Lhat (d) values at the same scale (Fig. 4). In addition, elevated CO_2_ concentration increased the scale range of regular distribution from 50 μm to 180 μm on the adaxial surface (Fig. 4a), while the scale range of regular distribution on the abaxial surface was decreased from 160 μm to 100 μm (Fig. 4b). In general, this enhanced CO_2_ concentration effect on the spatial distribution pattern of stomata was greater on the adaxial surface than the abaxial surface of soybean leaves.

### CO_2_ effects on leaf anatomic characteristics

**Fig. 5:**
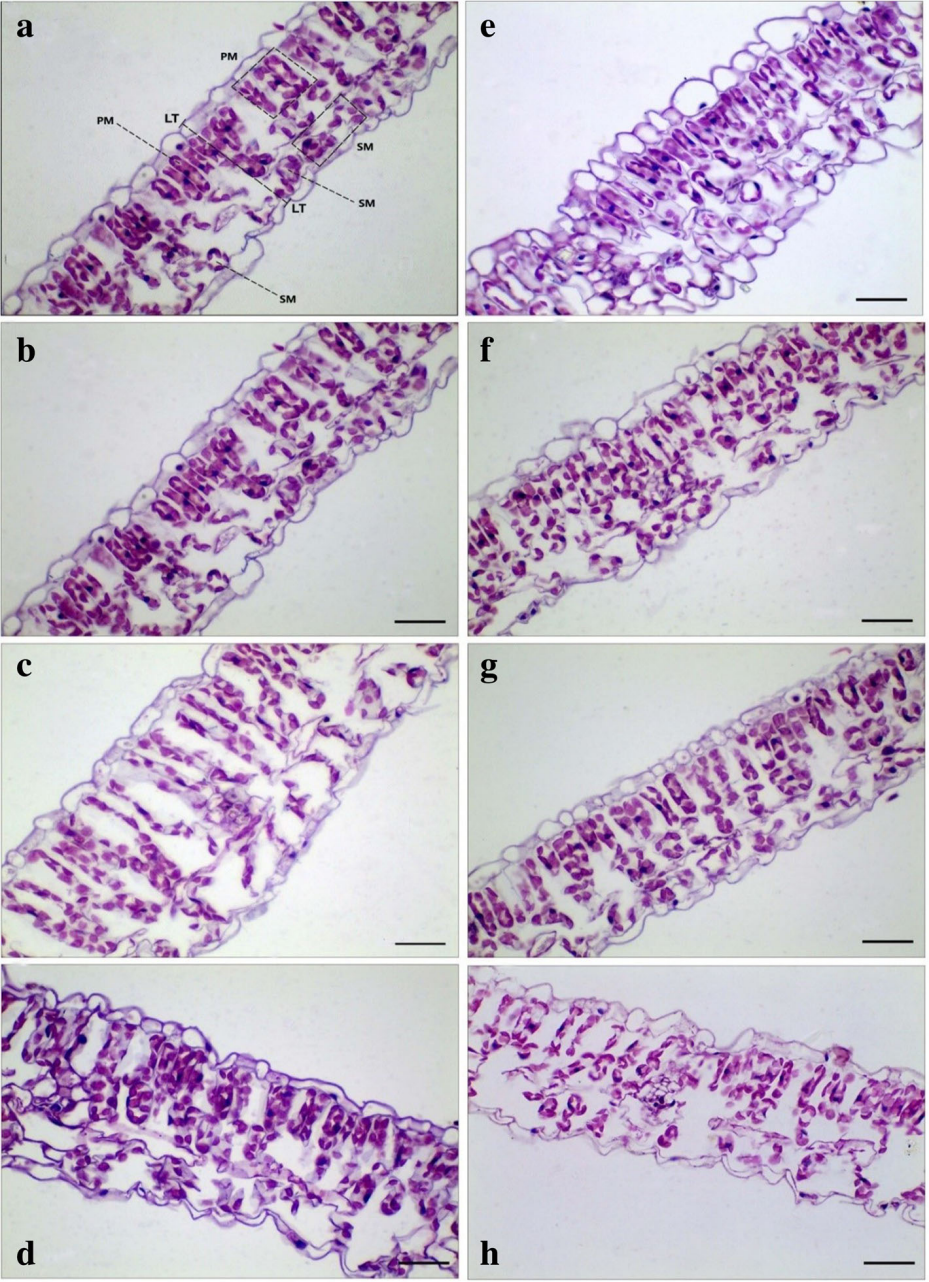
Light micrographs of cross-section through leaves of soybean. Note that cross-section micrographs show leaf thickness (LT), palisade mesophyll (PM), and spongy mesophyll (SM) of soybean leaves grown at ambient (**a**-**b**) and elevated CO_2_ concentrations (c-h). Bar = 50 μm

Elevated CO_2_ concentration significantly increased cell length, whereas decreased cell width of palisade mesophyll (PM) (*p* < 0.05; Table 3; Fig. 5). Elevating CO_2_ concentration from 400 ppm to 1000 ppm made the cell length of palisade layer increased from about 36 μm to 42 μm (Table 3). Relative to ambient CO_2_ concentration, the cell size of palisade layer was also significantly affected by elevated CO_2_ concentration (Fig. 5). Enhancing CO_2_ concentration from 400 ppm to 1000 ppm resulted in increases of cell area and cell perimeter by about 10 and 20%, mainly due to the larger cell length of PM. Elevated CO_2_ concentration from 400 ppm to 1000 ppm caused a decrease in the cell width of palisade by 23% (Table 3). Moreover, the cell length of spongy mesophyll (SM) was substantially enhanced by 32%, and thus the cell area was increased by 25% with elevating CO_2_ concentration of 1000 ppm. In addition, elevated CO_2_ concentration significantly affected both the thickness (LT) and the palisade/ spongy ratio of soybean leaves (*p* < 0.05; Fig. 5).

**Table 3:** Effects of elevated CO_2_ concentrations on the morphological and anatomical traits of soybean

Parameters	CO_2_ concentrations (ppm)							*p* value
	400	600	800	1000	1200	1400	1600	
Palisade layer								
Cell length (μm)	35.69 ± 3.04 (c)	39.14 ± 3.43 (ab)	38.59 ± 34.46 (b)	42.03 ± 5.23 (a)	33.38 ± 6.24 (d)	29.76 ± 4.47 (e)	41.15 ± 7.35 (a)	*p* < 0.001
Cell width (μm)	9.35 ± 1.76 (b)	8.93 ± 1.78 (b)	7.90 ± 31.22 (de)	9.00 ± 1.50 (bc)	7.25 ± 1.29 (e)	10.40 ± 1.18 (a)	8.13 ± 2.00 (cd)	*p* < 0.001
Cell area (μm^2^)	303.3 ± 39.6 (b)	348.7 ± 39.4 (a)	275.0 ± 76.2 (c)	328.0 ± 80.9 (ab)	213.1 ± 46.3 (d)	232.1 ± 60.5 (d)	303.5 ± 88.9 (bc)	*p* < 0.001
Cell perimeter (μm)	83.0 ± 6.4 (c)	95.1 ± 12.6 (b)	80.6 ± 9.9 (c)	99.3 ± 12.7 (ab)	71.4 ± 9.4 (d)	67.6 ± 10.2 (d)	100.9 ± 19.3 (a)	*p* < 0.001
Spongy layer								
Cell length (μm)	20.72 ± 2.43 (cd)	24.85 ± 4.00 (ab)	22.61 ± 5.45 (bc)	27.36 ± 7.75 (a)	19.87 ± 3.11 (d)	20.15 ± 2.87 (d)	20.07 ± 5.11 (d)	*p* = 0.009
Cell width (μm)	11.91 ± 3.13 (b)	12.04 ± 3.75 (a)	9.39 ± 5.44 (d)	10.54 ± 3.71 (ac)	8.28 ± 2.14 (e)	12.68 ± 2.15 (ad)	8.46 ± 2.40 (bef)	*p* < 0.001
Cell area (μm^2^)	207.8 ± 57.6 (b)	265.3 ± 88.4 (a)	192.0 ± 64.5 (b)	258.9 ± 24.5 (a)	150.0 ± 43.8 (c)	215.6 ± 53.0 (b)	177.2 ± 67.7 (b)	*p* = 0.010
Cell perimeter (μm)	86.2 ± 13.2 (bc)	76.88 ± 13.8 (a)	60.6 ± 13.3 (c)	71.4 ± 21.7 (ab)	55.5 ± 12.0 (d)	55.6 ± 13.7 (d)	63.1 ± 15.5 (bcd)	*p* = 0.020
Palisade/Spongy ratio	1.88 ± 0.68 (b)	2.52 ± 0.23 (a)	2.59 ± 0.64 (ab)	2.77 ± 0.54 (a)	2.13 ± 0.60 (abc)	1.90 ± 0.49 (bc)	1.75 ± 0.25 (c)	*p* < 0.031
Leaf thickness (μm)	103.5 ± 11.0 (c)	126.9 ± 9.9 (a)	121.5 ± 9.9 (b)	125.4 ± 12.7 (b)	108.7 ± 14.1 (c)	108.0 ± 12.2 (c)	106.9 ± 11.1 (c)	*p* = 0.001

### CO_2_ effects on tissue carbon and nitrogen

Elevated CO_2_ concentration dramatically affected tissue carbon (C) and nitrogen (N) as well as C/N of soybean plants (Table 4). Specifically, increasing CO_2_ concentration from 400 ppm to 1000 ppm substantially decreased C concentrations of leaf and stem (*p* < 0.001), whereas the root C content was significantly increased by 5% from 354.8 mg g^− 1^ to 371.2 mg g^− 1^ with further increasing CO_2_ concentration from 1000 ppm to 1600 ppm (*p* < 0.001). Moreover, elevated CO_2_ concentration enhanced the N content of stem and root (Table 4), while the leaf N was significantly decreased from 32.0 mg g^− 1^ to 30.8 mg g^− 1^ with increasing CO_2_ concentration from 400 ppm to 1200 ppm (*p* < 0.001; Table 4). Elevated CO_2_ concentration decreased the tissue C/N ratio due mainly to the increased N and decreased C in stems and roots (*p* < 0.001; Table 4). In addition, enhancing CO_2_ concentration from 400 ppm to 800 ppm slightly increased the leaf, stem, and total TNC by 12.8, 4.9, and 5.9%, whereas the TNC in leaves and stems were dramatically reduced with further increasing CO_2_ concentration from 800 ppm to 1600 ppm (Table 5).

**Table 4:** Effects of elevated CO_2_ concentrations on carbon and nitrogen contents of soybean

Element (mg g^−1^ DW)		CO_2_ concentrations (ppm)							*p* value
		400	600	800	1000	1200	1400	1600	
Leaf	C	403.6 ± 0.33(a)	386.14 ± 0.23(d)	390.54 ± 0.24 (d)	373.66 ± 12.73 (c)	393.30 ± 0.70 (bd)	392.07 ± 0.50 (bd)	395.71 ± 0.37(ab)	*p* < 0.001
	N	32.04 ± 0.12 (bc)	30.56 ± 0.22 (d)	33.56 ± 0.16 (a)	31.61 ± 1.00 (c)	34.04 ± 0.12 (a)	32.61 ± 0.07 (b)	30.83 ± 0.2 (d)	*p* < 0.001
	C/N	12.60 ± 0.05 (b)	12.64 ± 0.10 (b)	11.64 ± 0.06 (e)	11.82 ± 0.03 (d)	11.55 ± 0.03 (e)	12.02 ± 0.04 (c)	12.84 ± 0.08 (a)	*p* < 0.001
Stem	C	375.71 ± 0.66 (a)	358.33 ± 0.47 (b)	348.84 ± 0.17(d)	347.86 ± 0.20 (e)	335.51 ± 0.05 (g)	339.93 ± 1.01(f)	359.40 ± 0.30 (c)	*p* ***<*** 0.001
	N	26.47 ± 0.14(g)	32.58 ± 0.19 (f)	43.24 ± 0.25 (b)	45.85 ± 0.41 (a)	37.51 ± 0.10 (d)	42.24 ± 0.12 (c)	36.94 ± 0.20 (e)	*p* < 0.001
	C/N	14.19 ± 0.09 (a)	10.10 ± 0.06 (b)	8.07 ± 0.05 (e)	7.59 ± 0.07 (f)	8.95 ± 0.023.6 (d)	8.05 ± 0.04 (e)	9.73 ± 0.06 (c)	*p* < 0.001
Root	C	352.38 ± 0.13 (cd)	347.81 ± 0.42 (d)	350.68 ± 0.18 (cd)	354.78 ± 0.52 (c)	376.80 ± 0.62 (ab)	378.59 ± 2.97 (a)	371.19 ± 8.64 (b)	*p* < 0.001
	N	26.08 ± 0.29 (c)	34.40 ± 0.19 (b)	38.35 ± 0.09 (a)	37.70 ± 0.10 (a)	36.79 ± 0.25(a)	37.57 ± 0.33 (a)	33.59 ± 3.33 (b)	*p* < 0.001
	C/N	13.51 ± 0.15 (a)	10.11 ± 0.06 (c)	9.14 ± 0.02 (d)	9.41 ± 0.03 (d)	10.24 ± 0.08 (c)	10.07 ± 0.01 (c)	11.10 ± 0.81 (b)	*p* < 0.001

**Table 5:** Effects of CO_2_ concentrations on soluble sugars and starch concentrations of soybean

Non-structural carbohydrate (mg · g^−1^)		CO_2_ concentrations (ppm)							*p* value
		400	600	800	1000	1200	1400	1600	
Root	Soluble sugar	58.4 ± 5.0(bc)	96.9 ± 5.7(a)	61.2 ± 5.3(b)	40.1 ± 2.5(d)	63.4 ± 2.0(b)	65.2 ± 3.3(b)	52.1 ± 5.0(c)	*p* < 0.001
	Starch	26.8 ± 3.3(a)	17.2 ± 9.6(b)	25.6 ± 2.3(a)	18.1 ± 2.3(b)	18.4 ± 1.1(b)	20.16 ± 0.96(ab)	13.3 ± 1.5(b)	*p* = 0.014
	TNC	85.3 ± 7.8(b)	114.1 ± 14.8(a)	86.8 ± 6.5(b)	58.1 ± 2.6(c)	81.8 ± 3.0(b)	85.4 ± 3.9(b)	65.4 ± 6.5(c)	*p* < 0.001
Stem	Soluble sugar	51.1 ± 6.4(a)	59.7 ± 4.9(a)	55.8 ± 4.3(a)	64.5 ± 7.4(a)	54.2 ± 3.5(a)	47.2 ± 1.2(a)	53.3 ± 5.1(a)	*p* ***>*** 0.05
	Starch	16.2 ± 5.8(bc)	20.2 ± 2.7(ab)	20.2 ± 4.1(ab)	11.7 ± 0.3(c)	23.2 ± 4.1(a)	16.2 ± 0.7(bc)	10.9 ± 3.9(c)	*p* < 0.007
	TNC	67.3 ± 8.3(a)	79.9 ± 7.5(a)	75.9 ± 4.7(a)	76.2 ± 7.6(a)	77.4 ± 3.6(a)	63.4 ± 1.7(a)	64.2 ± 8.7(a)	*p* ***>*** 0.05
Leaf	Soluble sugar	65.5 ± 3.9(a)	30.5 ± 5.0(b)	72.6 ± 13.8(a)	59.0 ± 13.3(a)	27.4 ± 1.7(b)	29.7 ± 7.6(b)	22.6 ± 2.7(b)	*p* < 0.001
	Starch	17.9 ± 7.8(c)	26.0 ± 5.3(ab)	14.9 ± 2.0(c)	27.5 ± 4.4(a)	16.9 ± 5.0(c)	15.8 ± 1.7(c)	19.4 ± 1.9(bc)	*p* = 0.022
	TNC	83.4 ± 7.1(a)	56.5 ± 1.5(b)	87.5 ± 15.5(a)	86.5 ± 9.5(a)	44.3 ± 6.4(b)	45.5 ± 5.9(b)	42.0 ± 3.3(b)	*p* < 0.001
Total	Soluble sugar	182 ± 3(abc)	196 ± 27(ab)	212 ± 41 (a)	164 ± 11(bcd)	145 ± 30(cd)	142 ± 10(d)	128 ± 9(d)	*p* = 0.004
	Starch	61.0 ± 11.3(a)	63.4 ± 10.4(a)	60.7 ± 4.4(a)	57.3 ± 6.5(a)	58.5 ± 8.2(a)	52.2 ± 2.4(ab)	43.5 ± 6.4(b)	*p* < 0.001
	TNC	236 ± 7(ab)	250 ± 19(a)	250 ± 16(a)	221 ± 5(bc)	203 ± 27(c)	194 ± 8(cd)	171 ± 15(d)	*p* > 0.05

### Relationships among photosynthesis, leaf structure, non-structural carbohydrates, and nitrogen content

**Fig. 6:**
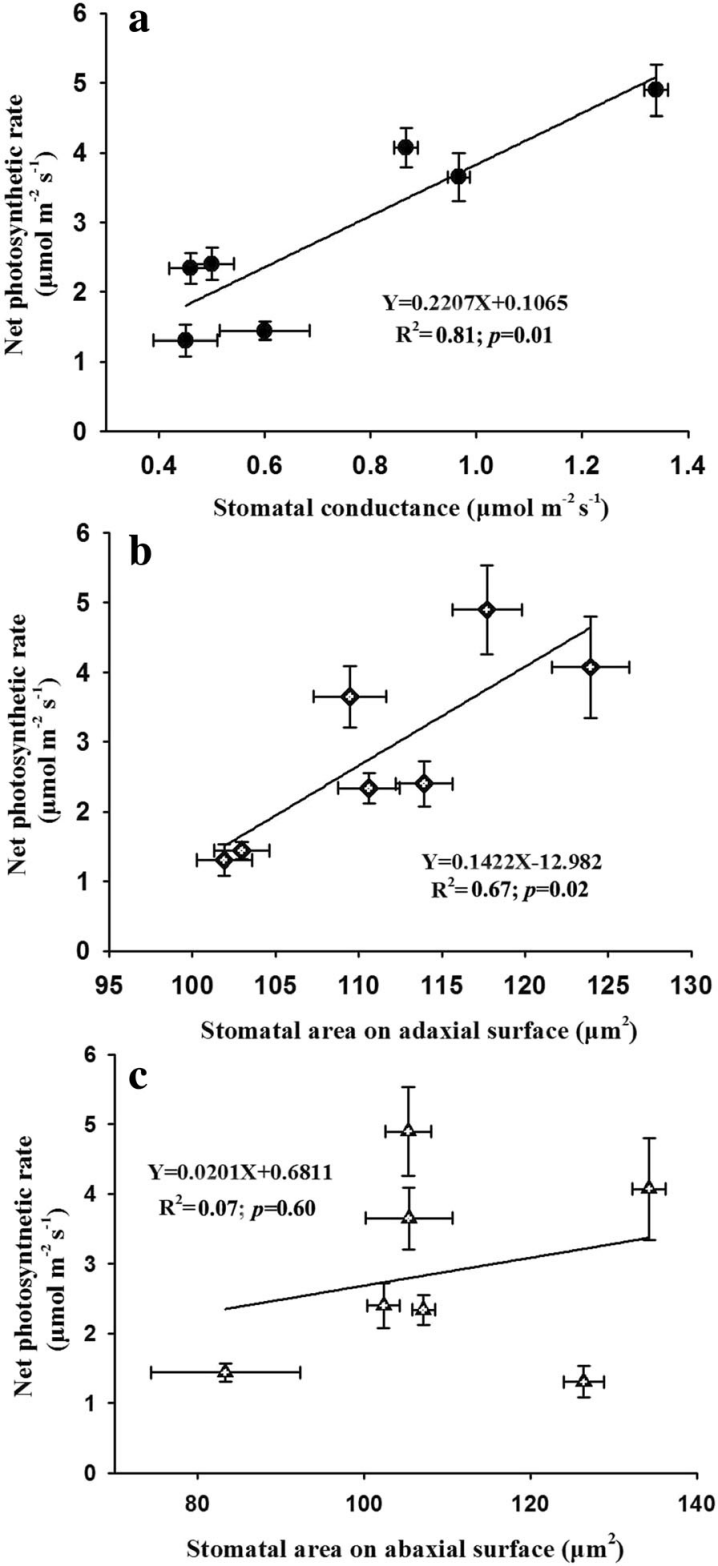
Effects of elevated CO_2_ concentrations on the relationships between net photosynthetic rate and stomatal conductance (**a**) as well as stomatal area (**b** and **c**) of soybean plants

**Fig. 7:**
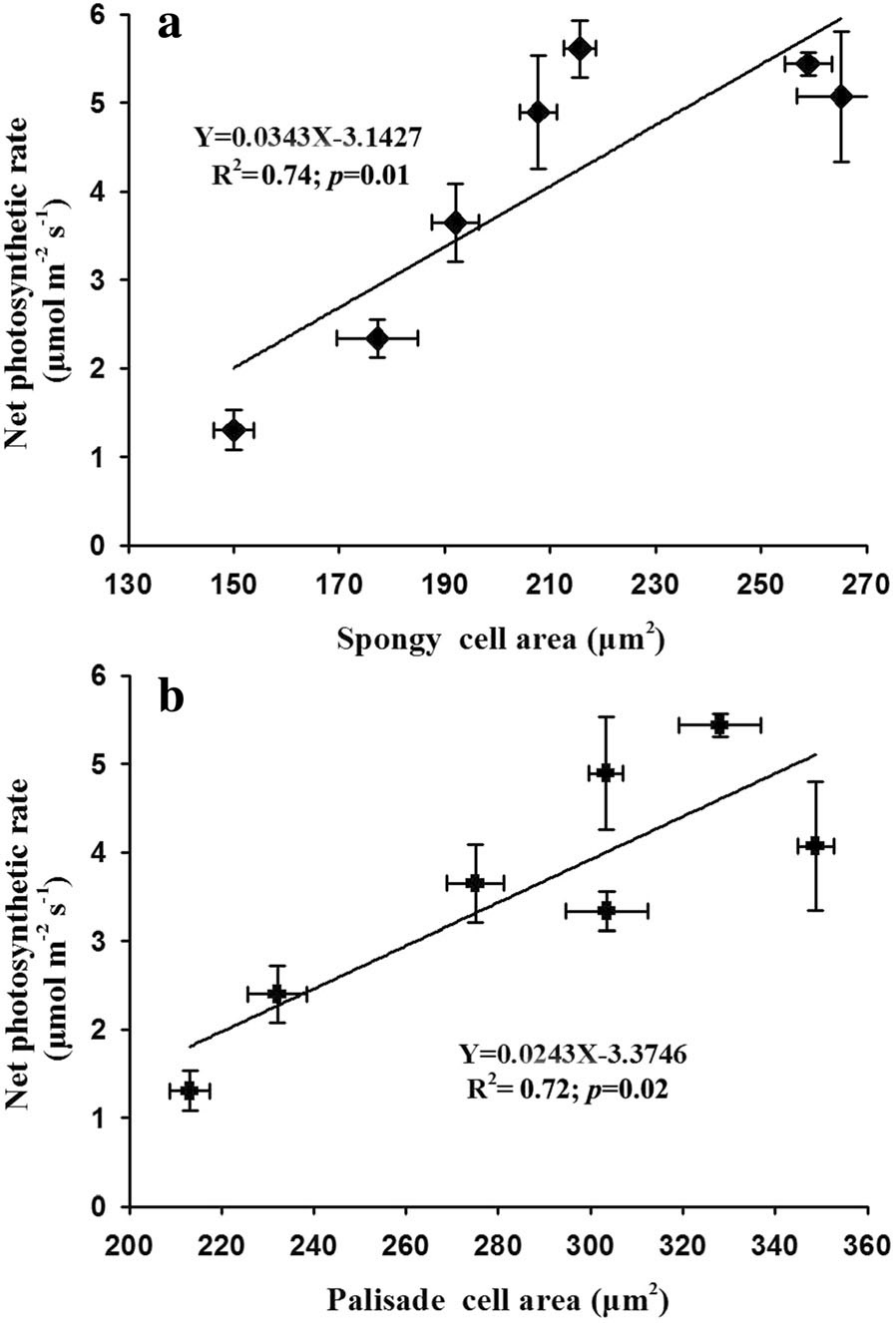
Effects of elevated CO_2_ concentrations on the relationships between net photosynthetic rate and spongy cell area (**a**) or palisade cell area (**b**) of soybean plants

**Fig. 8:**
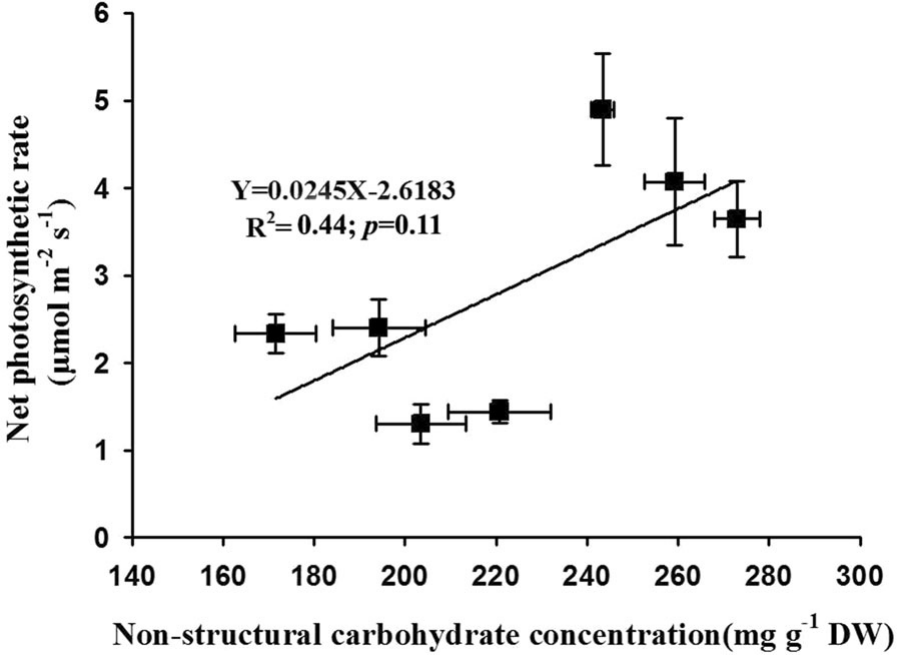
Effects of elevated CO_2_ concentrations on the relationships between net photosynthetic rate and non-structural carbohydrate concentration of soybean plants

We estimated the relationships between photosynthesis and stomatal conductance as well as photosynthesis and stomatal area and found that leaf photosynthesis was increased linearly by the enhancement of stomatal conductance and stomatal area on the adaxial surface with R^2^ values of 0.81 (*p* = 0.01) and 0.67 (*p* = 0.02), respectively (Fig. 6a-b). In contrast to the stomatal area on the adaxial surface, we found no linear relationship between leaf photosynthesis and stomatal area on the abaxial surface of soybean plants (R^2^ = 0.07, *p* = 0.60; Fig. 6c). Moreover, we also found that leaf photosynthesis was linearly increased by the cell enlargement of spongy and palisade tissues with R^2^ values of 0.74 (*p* = 0.01) for spongy cell area and 0.72 (*p* = 0.02) for palisade cell area, respectively (Fig. 7). In addition to leaf structure, we also evaluated the relationship among leaf photosynthesis, carbohydrates and nitrogen content. We found a positive but not significative relationship between leaf photosynthesis and non-structural carbohydrate content following a linear equation (R^2^ = 0.44, *p* = 0.11; Fig. 8).

## Discussion

### Down-regulation of leaf photosynthesis under elevated atmospheric CO_2_ concentrations

It is demonstrated that elevated CO_2_ concentration generally stimulates plant growth and enhanced crop yield through the CO_2_ fertilization effect [17, 18], whereby augmented atmosphere CO_2_ concentration can directly boost carboxylation in the Calvin-Benson-Bassham cycle and competitively inhibit dark respiration and photorespiration [13, 16]. By contrast, several studies claim that some plants may develop an adverse response through a process known as “down-regulation” of photosynthesis when plants exposed to higher CO_2_ concentration beyond certain thresholds [4, 26, 28]. We also found a negative quadratic relationship between leaf photosynthesis and CO_2_ concentration (R^2^ = 0.83; Fig. 1), indicating down-regulation of leaf photosynthesis did occur when soybean plants subjected to enhanced CO_2_ concentrations. This down-regulation of leaf photosynthesis may be caused by various limiting factors such as lower Rubisco concentration and activity [29, 30, 32, 34] reduced stomatal conductance [15, 68, 69], and excessive carbohydrates accumulation in leaves [29, 36–38].

Further analysis showed that leaf biochemical and photochemical efficiency might play a pivotal role in the down-regulation of leaf photosynthesis in the current study. Our results showed that the maximum carboxylation rate of Rubisco (*V*_cmax_) and the maximum capacity of electron transport RuBP regeneration (*J*_max_) were dramatically decreased by elevated CO_2_ concentrations, suggesting that enhanced CO_2_ concentrations may affect both the light and dark reactions of photosynthesis. Moreover, our results also suggested that elevated CO_2_ may have greater impacts on carboxylation processes than the photochemical processes as indicated by the rapidly decreased *V*_cmax_/*J*_max_ ratio beyond the optimal CO_2_ concentration of about 670 ppm (Fig. 2). Therefore, the lower carboxylation and photochemical efficiency as evidenced by the declines of the *V*_cmax_ and *J*_max_ values as well as the *V*_cmax_/*J*_max_ ratio at high CO_2_ concentrations may explain the negative CO_2_ effects on leaf photosynthesis of soybean plants as observed in the current study. Additionally, it is important to note that dark respiration increased with the change of CO_2_ from 400 ppm to 900 ppm, which may also contribute to the down-regulation of leaf photosynthesis. However, dark respiration started to decrease when the CO_2_ concentration is beyond 900 ppm which offsets the effects of *V*_cmax_ and *J*_max_ in the down-regulation of photosynthesis.

### Stomatal diffusion processes explain the down-regulation of leaf photosynthesis

In addition to biochemical and photochemical processes, our results also showed that enhancing CO_2_ concentrations generally decreased stomatal density on both leaf surface, especially the stomatal density on the adaxial leaf surface was substantially decreased by about 50% with increasing CO_2_ concentration from 400 ppm to 1600 ppm (Table 1). This CO_2_-induced decrease of stomatal density may explain the down-regulation of leaf photosynthesis because stomatal density partially determines CO_2_ diffusion efficiency from atmosphere to mesophyll tissues [52–54] and well correlates with stomatal conductance [47], which is closely associated with leaf photosynthesis [42, 58, 59]. Meanwhile, elevated CO_2_ concentrations significantly decreased the total stomatal area per unit leaf area (stomatal area index) on both leaf sides, suggesting the CO_2_-induced stomatal closure may also contribute to the decline of leaf photosynthesis through reducing stomatal conductance at high CO_2_ concentration. Previous studies have claimed that elevated CO_2_ can reduce stomatal openness by changing concentrations of ion and organic solutes and depolarizing the water potential of cell membrane [47, 54, 59].

In the current study, we also found well-correlated relationships among leaf photosynthesis, stomatal conductance, and stomatal area (Fig. 6), confirming that this down-regulation of leaf photosynthesis may be attributed to the decline of stomatal conductance through reducing stomatal openness when soybean plants exposed to high CO_2_ concentrations. Additionally, the less regular stomatal distribution pattern on the adaxial leaf surface of soybean plants as evidenced by the larger Lhat (d) at higher CO_2_ concentrations may contribute to the decline of stomatal conductance through increasing the average distance of CO_2_ diffusion from stomata to chloroplasts [47, 63]. Overall, the fewer stomata and smaller stomatal pore aperture, as well as the more irregular spatial distribution patterns at high CO_2_ concentrations may partially explain the decline of stomatal conductance in the current study. Also, several recent studies have claimed that down-regulation of leaf photosynthesis is well associated with the declined stomatal conductance, which is mainly attributed to decreases of stomatal density and stomatal openness [21, 70, 71]. It should be noted that the declined *G*_s_ does not necessarily reduce leaf photosynthesis when plants exposed to elevated CO_2_ concentrations [24]. Nevertheless, the leaf photosynthesis-*G*_s_ relationship did follow a linear equation in the current study (R^2^ = 0.81), indicating that the decreased stomatal conductance under high CO_2_ concentrations contributed to leaf photosynthesis down-regulation of soybean plants.

### Down-regulation of leaf photosynthesis associates with anatomical structure of mesophyll tissues

In addition to stomatal traits, the down-regulation of leaf photosynthesis is also associated with changes in the anatomical structure of mesophyll tissues at the high CO_2_ concentration [72–74]. Our results showed that the cell area of palisade and spongy tissues were increased by 15 and 28% as CO_2_ concentration increased from 400 ppm to 600 ppm, while the cell area of both the palisade and spongy tissues were marginally declined with further increase of CO_2_ concentration (Table 3). This decreased cell area of mesophyll tissues is likely to explain the down-regulation of leaf photosynthesis, because the smaller mesophyll cells at higher CO_2_ concentration may lead to narrow space for accommodating fewer chloroplasts through constraining the cell expansion, and thus limit the carbon gain efficiency of plants [52–54]. Xu also found that the average cell area of mesophyll tissue was decreased by about 30% at higher CO_2_ concentration [60]. Interestingly, we also found linearly positive relationships between leaf photosynthesis and mesophyll cell area, confirming that the down-regulation of leaf photosynthesis may be partially due to the smaller total mesophyll size (palisade and spongy tissues) of soybean plants under high CO_2_ environments.

### Changes in leaf non-structural carbohydrates and nitrogen attribute to down-regulation of photosynthesis

It is well documented that the down-regulation of photosynthesis is usually associated with changes in leaf chemical composition such as the N availability deficit [32–34], the lower Rubisco concentration and activity [33, 34] as well as the source-sink imbalance due to carbohydrates accumulation in leaves under high CO_2_ concentration [29, 36–38]. Previous studies have demonstrated that elevated CO_2_ concentration enhances leaf C/N ratio mainly due to the decline of N concentration through a process known as “N dilution” [61]. Our results showed that the leaf N was significantly decreased with increasing CO_2_ concentration from 400 ppm to 1200 ppm (Table 4), which may also attribute to the down-regulation of leaf photosynthesis, because leaf N is closely related to photosynthetic enzymes such as Rubisco [17]. However, several previous studies have claimed that the Rubisco concentration and activity of plants were substantially reduced at high CO_2_ concentration, because leaf N was prior to enzymes relating to the metabolic processes of carbohydrates than invested to Rubisco when plants were exposed to high CO_2_ environments [75]. Furthermore, it is important to note that hexokinase is a key functional enzyme for mediating sugar sensing, and thus may contribute the down-regulation of photosynthesis through decreasing the Rubisco concentration with inhibiting the expression of photosynthetic genes [38, 62].

In addition to leaf N, the down-regulation of photosynthesis induced by elevated CO_2_ is also possibly attributed to the accumulation of carbohydrates in leaves when plants subjected to high CO_2_ environments for a long time period [29, 46, 63, 64]. Our results showed that the total non-structural carbohydrates in leaves (TNC) was dramatically declined at higher CO_2_ concentrations (Table 5), suggesting that the source-sink imbalance of carbohydrates should not be a limiting factor for the down-regulation of photosynthesis in the current study. Moreover, we also found a positive linear relationship between leaf photosynthesis and TNC (Fig. 8), which directly supported the above conclusion that the imbalance of carbohydrate concentration in the source and sink contributed little to the leaf photosynthesis down-regulation of soybean plants subjected to high CO_2_ concentrations.

## Conclusions

We found that the net photosynthesis rate of soybean was dramatically declined with elevated CO_2_ concentration from 400 ppm to 1600 ppm following a typical quadratic relationship. This down-regulation of leaf photosynthesis at higher CO_2_ concentrations can be attributed to the limiting effects on stomatal diffusion processes and nitrogen availability as well as the changes in the biochemical and photochemical efficiency of photosynthesis. Overall, our results suggest that the continuously increasing CO_2_ concentration in the future may lead to negative impacts on agricultural production through hurting crop growth and/or reducing crop yield. Nevertheless, most of the projections estimated the plant growth and crop production according to the earlier results from “doubling-CO_2_ experiments” with strong CO_2_ fertilization effect. Therefore, many current climate change models may underestimate the potential risk of climate change on agricultural production mainly due to the overestimated strong CO_2_ fertilization effect on plant growth and crop yield under future elevated atmospheric CO_2_ concentration and climate change.

## Methods

### Growth chamber experiments

We bought soybean seeds from the Wotu seed company in Hebei Province of China. We grew three plants in each pot (30 cm diameter × 50 cm long), then set up five pots in each of the seven walk-in environmental growth chambers for 90 days CO_2_ treatment, where the CO_2_ concentration was regulated to ambient concentration (400 ppm) or elevated concentrations (600, 800, 1000, 1200, 1400 and 1600 ppm). The ambient and elevated CO_2_ concentrations within the chambers were maintained through a CO_2_ tank containing high purity CO_2_ gas (99.99%) to avoid any hurt or pollution on winter wheat plants. All of the seven growth chambers were maintained with the same other environmental factors including relative humidity of 65%, photosynthetic photon flux density (PPFD) of 1000 μmol m^− 2^ s^− 1^, temperature of 25/21 °C (day/night), and 12-h photoperiod for the 90 days treatment. These winter wheat plants were fertilized with half-strength Hoagland’s solution twice weekly (150 mL per pot) and irrigated once daily with plain tap water (200 mL per pot) during the establishment and treatment periods of soybean plants under elevated CO_2_ concentrations.

### Measuring leaf gas exchange

We performed the measurements of leaf gas exchange at the end of the CO_2_ treatment period. We randomly selected one fully expanded leaf from each pot for leaf gas exchange measurement (*n* = 5) with a portable photosynthesis system (LI-6400XT; LICOR, Inc.). These selected leaves were firstly equilibrated at the corresponding growth CO_2_ levels with saturating PPFD of 1500 μmol photon m^− 2^ s^− 1^ and growth temperature of 25 °C. The portable photosynthesis system automatically controlled the CO_2_ concentrations in the cuvette using an injector system combined with a CO_2_ mixer. All of the measurements on leaf gas exchange were performed with the vapor pressure deficit (*VPD*) lower than 1.5 kPa to avoid moisture limitation. Then, the photosynthesis vs intercellular CO_2_ (*A*_n_-*C*_i_) curves were measured at cuvette chamber CO_2_ of 50, 100, 150, 200, 300, 400, 600, 800, 1000, 1200, 1400, and 1600 ppm. Data from *A*_n_-*C*_i_ curves were used to compare treatment effects on the light-saturated net photosynthetic rates (*A*_n_) at ambient or elevated CO_2_ of their growing condition. *A*_n_ estimation method was used to obtain the maximum carboxylation rate of Rubisco (*V*_cmax_), and the maximum capacity of electron transport mediated ribulose bisphosphate (RuBP) regeneration (*J*_max_) for each observed *A*_n_-*C*_i_ curve. Meanwhile, stomatal conductance (*G*_s_), intercellular CO_2_ concentration (*C*_i_), transpiration rate (*T*_r_), and dark respiration rate (*R*_d_) were also determined with the portable photosynthesis system (LI-6400XT; LICOR, Inc.). In addition, the leaf-level water use efficiency (*WUE*) was determined by the values of the net photosynthetic rate (*A*_n_) and transpiration rate (*T*_r_) according to the formula *WUE* = *A*_n_ / *T*_r_.

### Measuring morphological traits of individual stoma and spatial distribution pattern of stomata

We randomly selected five fully expanded ear leaves at the heading stage in each of the ambient and elevated CO_2_ concentration plots to determine the stomatal characteristics. We sampled impressions of stomata with colorless nail polish from the middle section of the adaxial and abaxial leaf surfaces. Firstly, the adaxial and abaxial leaf epidermis were carefully cleaned with degreased cotton balls and then smeared with nail varnish from the mid-area between the leaf edge and the central vein for half an hour. The thin film with stomatal impression (approximately 5 mm × 15 mm) was peeled off from the leaf surface and mounted on a glass slide, and immediately covered with a cover slip and lightly pressured with a fine-point tweezer [47, 63]. We photographed the stomatal features with a microscope (DM2500, Leica Corp, Germany) equipped with a digital camera (DFC 300-FX, Leica Corp, Germany), and then analyzed thirty separate fields of 0.16 mm in each leaf section. We also combined and counted the stomata on each surface for calculating stomatal density (SD) of the adaxial and abaxial surface, respectively [47]. Moreover, we randomly selected six digital photographs of the adaxial and abaxial surfaces to measure the stomatal length (SL), stomatal width (SW), stomatal area (SA) and stomatal perimeter (SP) using AutoCAD 2010 software. In addition, we calculated stomatal shape index (SSI), which is calculated by the function that shape index= $\frac{\sqrt{\mathrm{SA}}}{\ \mathrm{SP}}\times 100\%$, where SA is the stomatal area and SP is the stomatal perimeter. The stomatal area index (SAI) is defined as the total stomatal area per unit leaf area calculating as stomatal average density × stomatal area per stoma × 100%. In addition to stomatal density and pore traits, we also characterized the spatial distribution pattern of stomata for each image by digitizing the stomatal positions into a shape file in GIS with the ArcMap software [47]. The spatial distribution pattern of stomata on leaves was quantified using the Ripley’s K-function with generating the x and y coordinates of stomata for each image in GIS and then calculating the Lhat (d) value (the transformed K value) based on these stomatal coordinates using the R statistic software. We compared the Lhat (d) values at different scales (distances) for detecting the spatial distribution pattern of stomata with the upper and lower boundaries generated by the 95% confidence level with the Monte Carlo simulations of 100 replicates [47, 76]. In the current study, we only reported the spatial distribution patterns of stomata on the middle section of the leaves due to the large number of stomatal images of winter wheat leaves.

We snapped three leaf pieces (2 mm × 2 mm) from the middle section of each leaf and fixed them with 2.5% (v/v) glutaraldehyde (0.1 M phosphate buffer, pH 7.0) to visualize the changes in stomatal morphology among different CO_2_ concentrations. Firstly, we washed these leaf samples several times with buffer and fixed them in 1% (v/v) osmium tetroxide for three hours and these samples were dehydrated with an ethanol series. Then, these leaf samples were carefully coated with gold in a high-vacuum evaporation unit. Finally, we examined and photographed the morphological traits of stomata with a scanning electron microscopy (FEI Corp, USA).

### Measuring leaf anatomical structures

Changes in the leaf internal anatomy of the winter wheat plants exposed to different CO_2_ concentrations were examined with leaf cross-sections under a light microscopy [77]. These images of leaf cross-sections were collected from the middle section of leaves to observe and measure leaf anatomical features using Image J software (NIH, USA). We estimated leaf mesophyll thickness between epidermal layers at five points in each cross-section [78]. We also randomly selected 20 clear palisade layer cells and 20 sponge layer cells from each leaf cross-section image to measure cell length, cell width, cell area, and cell perimeter with an Auto CAD software.

### Analyzing leaf non-structural carbohydrates and nitrogen

We collected leaf samples from each pot as a replicate (*n* = 5 pots) for analyzing the non-structural carbohydrates. These sampled leaves were dried with an oven at 75 °C for 48 h to consistent weight, and then these samples were ground to fine powder for spectrophotometrically analyzing glucose, fructose, sucrose, and starch with a glucose kit [79]. Similarly, we also sampled plant tissues from each pot (n = 5 pots) for analyzing the total carbon (C) and nitrogen (N) in different plant tissues (leaf, stem, and root) with an elemental analyzer [80]. All of the analyses were expressed on a percentage dry matter basis.

### Analyzing data

We used the one-way ANOVA to analyze the effects of CO_2_ on the stomatal traits, soluble sugar and starch concentrations, carbon and nitrogen contents, as well as morphological and anatomical features. Two-way ANOVA was employed to test the effects of CO_2_ concentration and leaf surface position (abaxial vs. adaxial) on the morphological traits of stomata with statistically significant differences at *p* < 0.05 level. We also employed linear and non-linear regressions for estimating the relationships between CO_2_ concentration and other variables. The raw data from the leaf photosynthesis measurements were processed in Excel spreadsheets where the non-linear *A*_n_-*C*_i_ curve fitting was performed [81]. The net assimilation rate (*A*_n_) versus intercellular CO_2_ concentration (*A*_n_-*C*_i_ curve), was fitted to estimate the maximum carboxylation rate (*V*_cmax_), maximum electron transport rate (*J*_max_) based on the measurements of *A*_n_-*C*_i_ curves. In addition, linear and non-linear (quadratic equations) regressions were employed to examine the relationships between CO_2_ concentration and other variables.

## Abbreviations

*A*_n_: Net assimilation rate
*A*_n_-*C*_i_ curve: Net assimilation rate versus intercellular CO_2_ concentration curve
*C*_i_: intercellular CO_2_ concentration
*G*_s_: stomatal conductance
*J*_max_: Maximum electron transport rate
*R*_d_: dark respiration rate
*V*_cmax_: Maximum carboxylation rate
*VPD*: vapor pressure deficit
